# Non-coding RNAs in parasitic diseases: a bibliometric analysis of knowledge structure, research hotspots, and future directions

**DOI:** 10.3389/fcimb.2026.1841931

**Published:** 2026-07-31

**Authors:** Xin Wang, Yuan Zhang, Jingyuan Li, Feng Xue, Di Zhang, Na Li, Jie Zhang, Zengru Xie, Hailong Guo, Wen Zhao

**Affiliations:** 1 Department of Minimally Invasive Spine Surgery and Precision Orthopedics, The First Affiliated Hospital of Xinjiang Medical University, Urumqi, China; 2 Xinjiang Engineering Technology Research Center for Medicine-Industry Integration, Xinjiang Medical University, Urumqi, China; 3 Department of Orthopedics and Trauma, the First Affiliated Hospital of Xinjiang Medical University, Urumqi, China; 4 Medical Laboratory Center, The First Affiliated Hospital of Xinjiang Medical University, Urumqi, China; 5 Key Laboratory of High Incidence Disease Research in Xingjiang (Xinjiang Medical University), Ministry of Education, Urumqi, China; 6 Xinjiang Clinical Research Center for Orthopedics, Xinjiang Medical University, Urumqi, China

**Keywords:** bibliometrics, CiteSpace, extracellular vesicles, non−coding RNAs, parasitic diseases, research hotspots

## Abstract

**Background:**

Non-coding RNAs (ncRNAs) have emerged as critical regulators in host–parasite interactions, yet the rapid proliferation of publications has made it challenging to obtain a comprehensive, unbiased view of the field’s intellectual structure and evolving frontiers.

**Methods:**

We performed a bibliometric analysis of research articles on ncRNAs in parasitic diseases published from 2015 to 2025. A multi−database search was conducted across the Web of Science Core Collection, PubMed, and the Cochrane Library, yielding 432 records for annual publication trend analysis, with 409 original research articles from the Web of Science Core Collection used for bibliometric network analyses, including co−citation, collaboration networks, keyword clustering, burst detection, and dual−map overlay.

**Results:**

Annual publications grew steadily from 21 to 44 over the decade, with a peak of 51 in 2022. China ranked first in output (175 articles), while the United States showed the highest centrality (0.37). Author collaboration networks were sparse (density = 0.0032), indicating fragmented research efforts. Keyword clustering identified 11 clusters, with “liver” and “immune responses” as persistent core themes; “extracellular vesicles” emerged as a key hub after 2018. Citation burst analysis revealed a three−phase evolution—from molecular identification (2015–2019) to functional regulation and communication (2019–2021), and then to host–pathogen interactions and translational applications (2022–2025). The most highly cited references collectively established the “extracellular vesicles–ncRNA” paradigm, and persistently bursting references point toward clinical diagnostics. Circular RNAs and the NF−κB pathway appeared as emerging hotspots. Journal overlay mapping showed knowledge flowing from molecular biology toward veterinary and parasitology disciplines.

**Conclusions:**

The field is transitioning from fundamental discovery toward translational applications, with extracellular vesicle−encapsulated ncRNAs holding promise as biomarkers and therapeutic targets. Enhanced international collaboration and interdisciplinary integration are urgently needed to accelerate progress.

## Introduction

Parasitic diseases remain a formidable global health burden, with pathogens such as *Plasmodium*, *Schistosoma*, and *Leishmania* responsible for immense morbidity and mortality, particularly in low- and middle-income countries (Anisuzzaman et al., 2023). The complex life cycles of these parasites, their sophisticated immune evasion strategies, and the relentless emergence of drug resistance have exposed the limitations of research paradigms centered exclusively on protein−coding genes (Balahbib et al., 2025). In recent years, non−coding RNAs (ncRNAs)—including microRNAs (miRNAs), long non−coding RNAs (lncRNAs), and circular RNAs (circRNAs)—have fundamentally reshaped our understanding of the molecular landscape of host−parasite interactions (Psvv et al., 2025). These regulatory molecules orchestrate diverse biological processes essential for both parasite survival and host defense. Perhaps the most paradigm−shifting discovery is the ability of parasites to deliver functional ncRNAs to host cells via extracellular vesicles (EVs), a “cross−kingdom” communication mechanism that directly reprograms host gene expression to favor parasite persistence (Ding et al., 2019; Sais et al., 2024). This revelation has not only deepened our understanding of pathogenesis but has also opened unprecedented avenues for the development of non−invasive diagnostic biomarkers and innovative multi−target therapeutic strategies against these refractory infections (Li et al., 2025; Tian et al., 2025).

Despite these remarkable advances, the field faces a challenge of data richness but insight poverty. The exponential growth of ncRNA−related publications, fueled by high−throughput sequencing, has created a vast and fragmented literature that makes it difficult for individual researchers to maintain a comprehensive, unbiased perspective on the field’s intellectual structure, thematic evolution, and emerging frontiers. Traditional narrative reviews, while valuable, are inherently constrained by authors’ subjective expertise and selection bias, rendering them ill−suited for capturing the macro−level dynamics of a rapidly expanding interdisciplinary domain. Consequently, a critical knowledge gap persists: we lack a clear, data−driven framework to systematically organize this complex landscape, obscuring our ability to answer fundamental questions regarding the field’s foundational works, the trajectories of its research themes, and its most promising nascent directions.

Bibliometric analysis offers a robust and objective pathway to address this gap. By applying computational and statistical methods to large−scale publication metadata—including authorship, institutional affiliations, keywords, and citation patterns—this approach constructs empirical “science maps” that reveal the social, intellectual, and conceptual architecture of a research domain. Unlike traditional reviews, bibliometrics provides a panoramic, quantitative overview that can identify invisible collaboration networks, trace the evolution of research themes through keyword co−occurrence and clustering, and highlight influential works and emerging topics through citation and burst detection algorithms. Tools such as CiteSpace are specifically designed for this purpose, transforming abstract bibliographic relationships into interactive visualizations that enable researchers to navigate the scientific literature with enhanced clarity and strategic foresight.

In light of this, the present study employs a comprehensive bibliometric approach to systematically analyze the knowledge landscape, research focal points, and developmental trajectories in the field of ncRNAs in parasitic diseases over the period from 2015 to 2025. Through this analysis, we aim to characterize the annual publication growth and delineate the collaboration networks of key contributing countries, institutions, and authors; to map the evolution of research themes and identify current hotspots through keyword co−occurrence, clustering, and citation burst analysis; to elucidate the intellectual foundation of the field by examining the co−citation patterns of landmark studies; and to explore the flow of knowledge between disciplines through journal dual−map overlay analysis. Ultimately, this investigation seeks to provide a dynamic and objective blueprint of the parasitic ncRNA research field, highlighting critical knowledge gaps and offering data−driven insights that can help researchers navigate this promising and rapidly evolving area of science.

## 2 Data and methods

### 2.1 Data acquisition

This study conducted comprehensive searches in three databases: Web of Science Core Collection (SCI-Expanded), PubMed, and the Cochrane Library, covering the period from January 1, 2015 to December 31, 2025. The search strategy was constructed around two major themes—”parasites” and “non-coding RNAs”—using title field retrieval.

Taking the Web of Science database as an example, the full search string was: TI= (parasite OR “parasitic disease*” OR malaria OR plasmodium OR leishman* OR trypanosom* OR toxoplasm* OR babesia OR theileria OR amoeb* OR entamoeba OR naegleria OR acanthamoeba OR giardia* OR cryptosporid* OR trichomonas OR blastocystis OR microsporid* OR isospor* OR cyclospor* OR sarcocystis OR helminth* OR nematode* OR cestode* OR trematode* OR schistosom* OR bilharzia OR filaria* OR “lymphatic filariasis” OR elephantiasis OR ascari* OR trichuris OR whipworm OR strongyloides OR hookworm OR ancylostoma OR necator OR dracunculus OR anisakis OR angiostrongylus OR echinococc* OR hydatid* OR hymenolepis OR taenia OR cysticerc* OR clonorchis OR opisthorchis OR fasciola OR paragonimus) AND (microRNA OR miRNA OR miR-* OR “non-coding RNA” OR ncRNA OR lncRNA OR “long noncoding RNA” OR “long non-coding RNA” OR circRNA OR “circular RNA” OR siRNA OR piRNA OR snoRNA OR snRNA OR “small interfering RNA” OR “PIWI-interacting RNA” OR “small nucleolar RNA” OR “small nuclear RNA” OR exosomal RNA OR “extracellular RNA” OR “cell-free RNA”).

For PubMed and the Cochrane Library, the search strategies were adapted to each database’s syntax, also using title field retrieval. All retrieved records were imported into NoteExpress for deduplication and preliminary screening. Given that CiteSpace requires complete citation data for cluster analysis and collaboration network construction, and the export formats from PubMed and the Cochrane Library do not include standardized citation information, only Web of Science data were used for subsequent network analyses. Search results from PubMed and the Cochrane Library were used for supplementary screening and publication count calculation, ensuring the most comprehensive coverage of research output in this field.

### 2.2 Inclusion and exclusion criteria

Inclusion criteria were peer−reviewed original research articles focusing on parasites and non−coding RNAs, published between January 1, 2015 and December 31, 2025, with no language restrictions.

The following document types were excluded: REVIEW, MEETING ABSTRACT, EDITORIAL MATERIAL, CORRECTION, LETTER, RETRACTED PUBLICATION, CONFERENCE PROCEEDINGS, DUPLICATE PUBLICATION, LECTURE, META−ANALYSIS, RETRACTION NOTICE, SCOPING REVIEW, and SYSTEMATIC REVIEW. Duplicate records were removed after deduplication. Additionally, articles clearly irrelevant to the research topic or with incomplete data were excluded.

**Figure 1:**
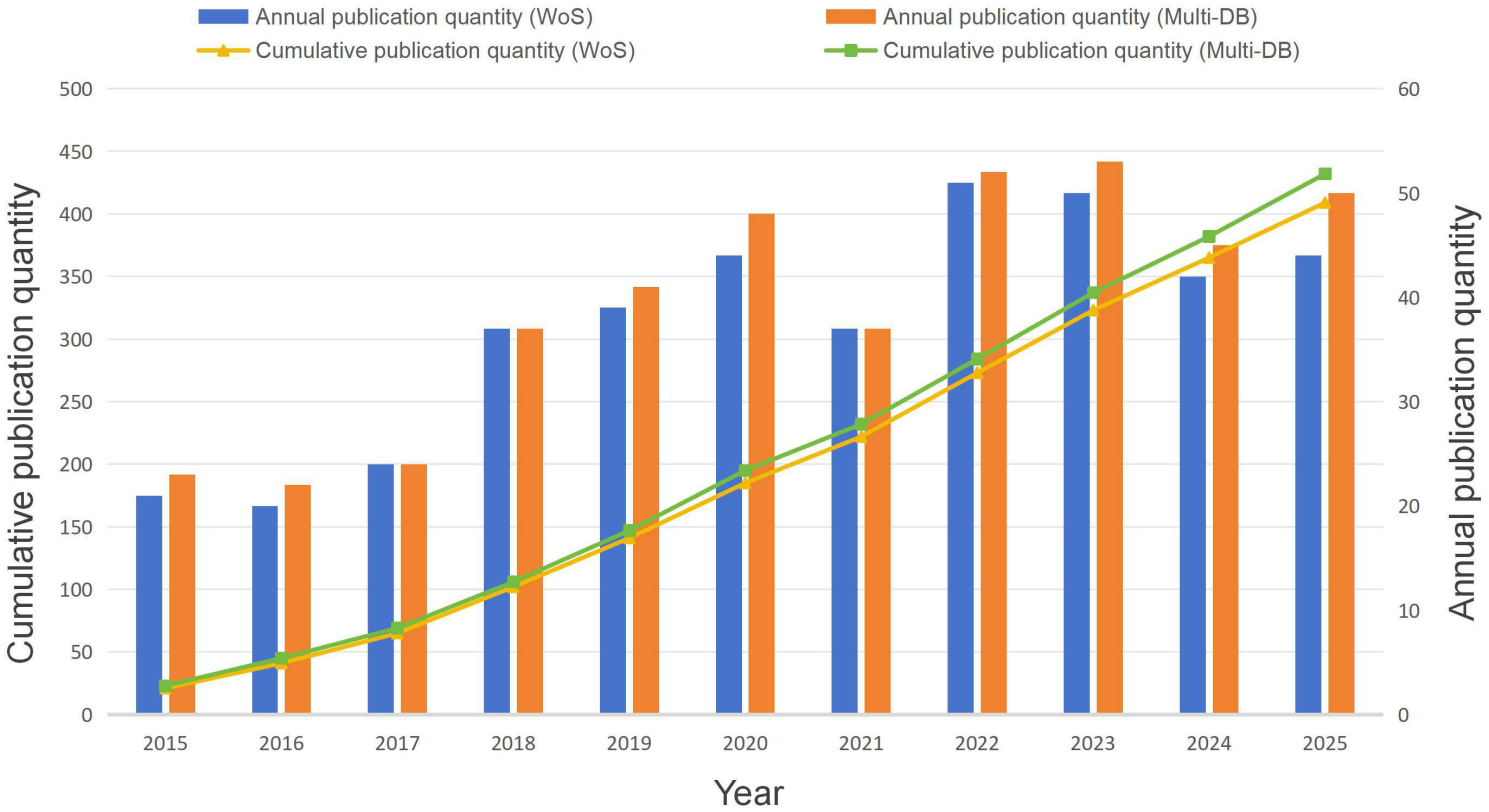
Annual and cumulative publication trends of ncRNA research in parasitic diseases (2015–2025). Bars represent annual publication counts; lines represent cumulative totals. WoS: records from the Web of Science Core Collection (n=409). Multi−DB: records from all three searched databases—Web of Science, PubMed, and the Cochrane Library (n=432).

Following this screening process, 432 unique records were identified across the three databases for publication trend analysis. The annual distribution of these 432 records is presented in **Figure 1** and summarized in **Table 1**. Among these, 409 eligible original research articles from the Web of Science Core Collection were finally included for CiteSpace−based bibliometric network analysis.

**Table 1:** Top 10 countries by publication volume and centrality.

Rank	Publication volume	Centrality	Publication year	Country
1	175	0.17	2015	PEOPLES R CHINA
2	64	0.37	2015	USA
3	39	0.02	2015	BRAZIL
4	31	0.03	2018	IRAN
5	29	0.03	2015	INDIA
6	27	0.15	2015	ENGLAND
7	21	0.34	2015	GERMANY
8	16	0.08	2015	FRANCE
9	12	0.07	2015	TURKIYE
10	12	0.01	2015	EGYPT

### 2.3 Analytical framework

CiteSpace 6.3.R1 (64-bit) was used for bibliometric visualization analysis. The main analytical modules included collaboration network analysis (countries, institutions, authors), keyword co-occurrence and cluster analysis, keyword burst detection, reference co-citation analysis, and journal dual-map overlay. CiteSpace is one of the most widely used bibliometric visualization tools, capable of effectively identifying research hotspots, frontiers, and knowledge structures in various fields.

Parameter settings were as follows: time span from 2015 to 2025 with 1-year time slices; node types set to country, institution, author, keyword, and reference according to the specific analysis; the g-index (k=25) was selected as the thresholding criterion; and network pruning was performed using the Pathfinder algorithm. The g-index is a bibliometric indicator that assigns greater weight to highly cited publications and performs better than the traditional h-index in identifying core literature; k=25 is the default g-index setting in CiteSpace, balancing network connectivity with manageable node density. The Pathfinder algorithm highlights core network structures by removing redundant connections, thereby improving visualization and readability. All other parameters remained at their default settings. Data analysis was conducted from February to June 2026.

## 3 Results

### 3.1 Literature retrieval and publication trends

**Figure 2:**
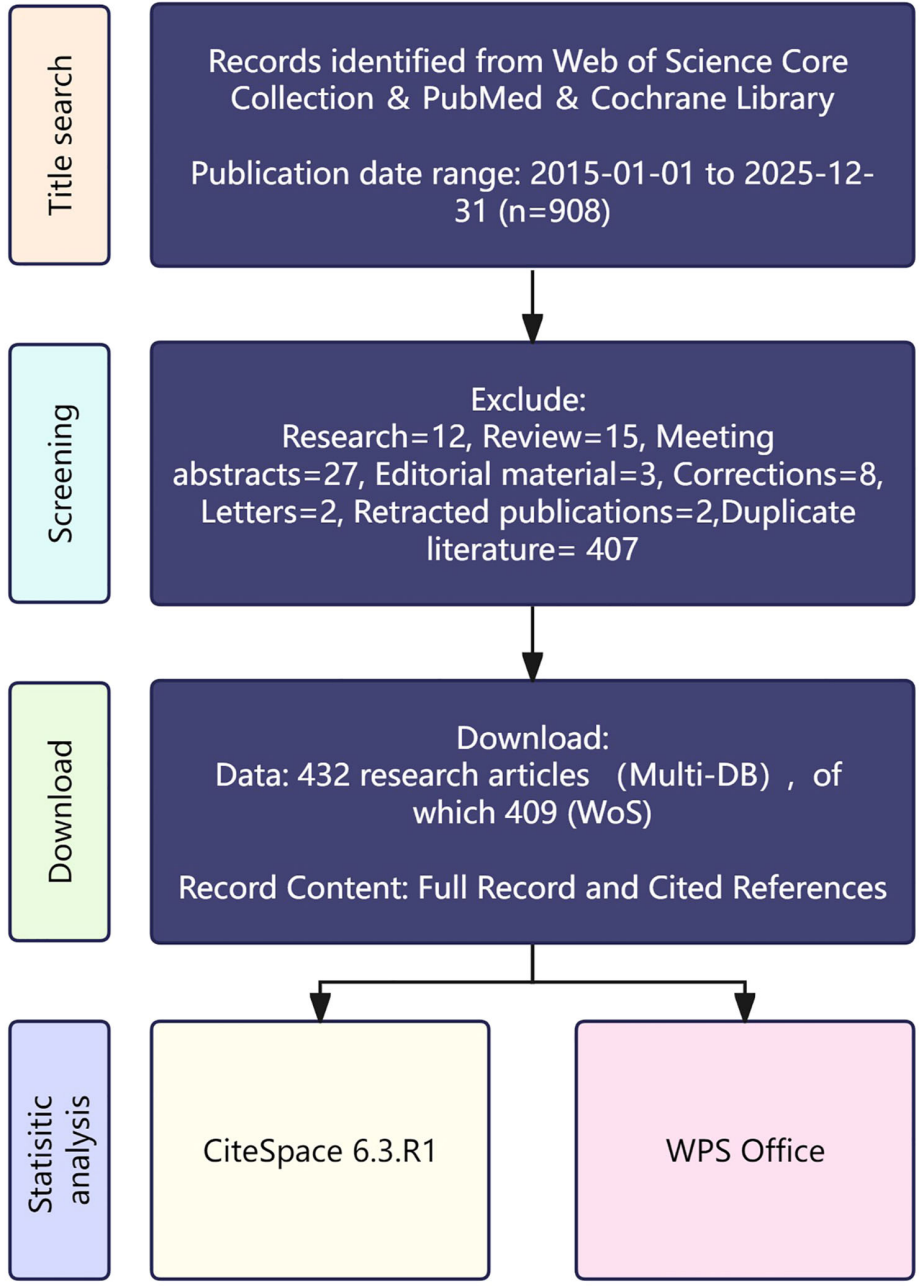
Flow diagram of the literature screening and selection process. Of 908 records identified from WoS (Web of Science Core Collection), PubMed, and Cochrane Library, 432 unique records remained after deduplication and exclusion of non−original articles, of which 409 WoS original research articles were included for CiteSpace analysis. Multi−DB: combined records from the three databases.

A total of 432 unique records were retrieved from the three databases after deduplication. After exclusion of reviews, meeting abstracts, conference proceedings, systematic reviews, and other non−original article types, 409 original research articles from the Web of Science Core Collection were finally included for bibliometric network analysis (**Figure 2**).

From 2015 to 2025, annual publications exhibited a fluctuating upward trend based on the 432 records identified across all three databases. The annual count increased from 23 in 2015 to 48 in 2020, declined slightly to 37 in 2021, and then rebounded to reach peaks of 52 in 2022 and 53 in 2023—the highest annual output during the study period. This was followed by 45 articles in 2024 and 50 articles in 2025. Cumulative publications grew steadily from 23 in 2015 to 106 in 2018, surpassed 200 in 2021 (232 articles), and reached 432 by the end of 2025 (**Figure 1**).

### 3.2 National, institutional, and author collaboration networks

**Figure 3:**
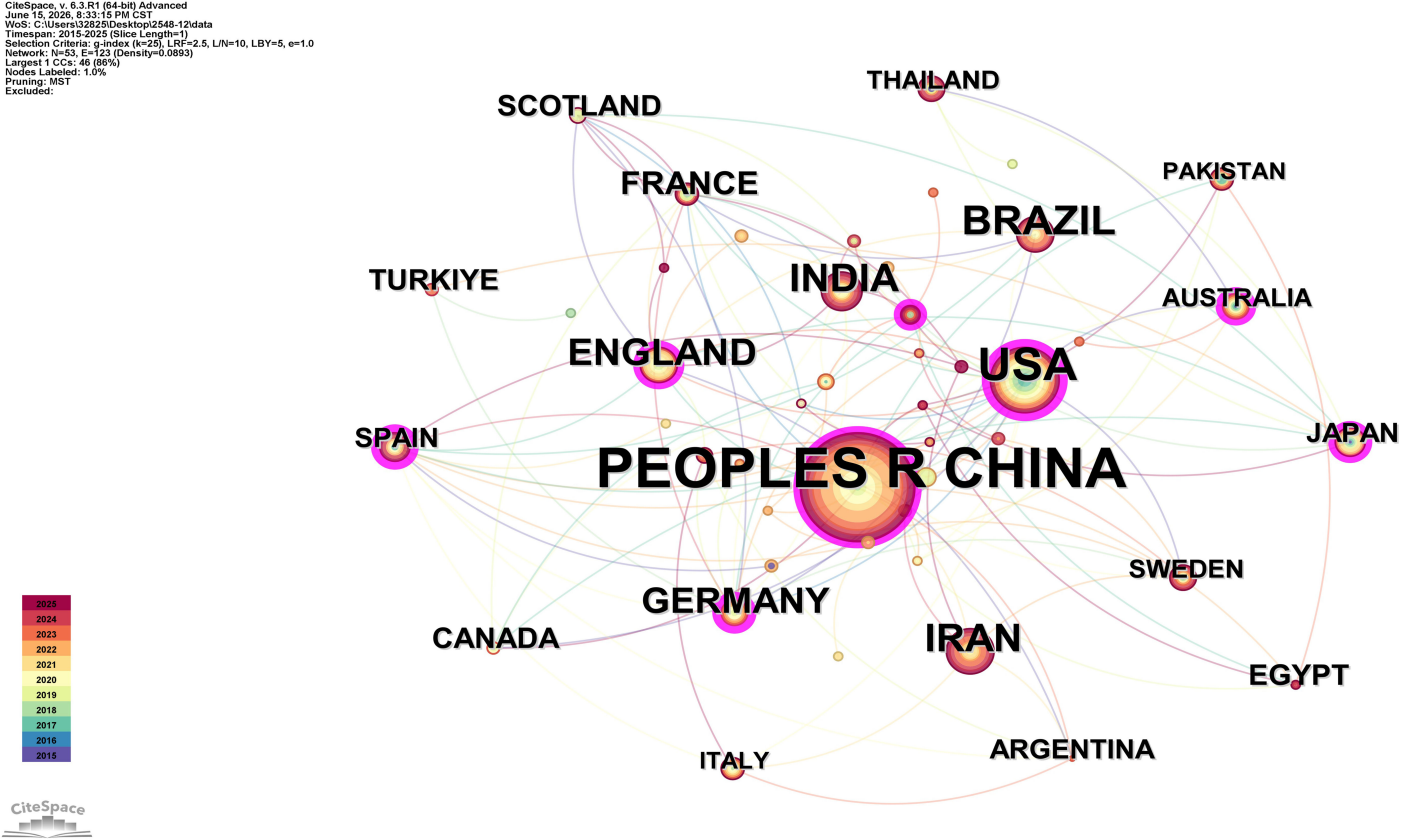
Country co−occurrence network. Nodes represent countries/regions; node size reflects publication volume; link thickness represents collaboration strength. The network consists of 53 nodes and 123 edges (density=0.0893).

The country co-occurrence network consists of 53 nodes and 123 links, with a density of 0.0893 (**Table 1**; **Figure 3**). The top five countries by output were China (175), the United States (64), Brazil (39), Iran (31), and India (29), with China ranking first by a substantial margin. The top five by centrality were the United States (0.37), Germany (0.34), Spain (0.29), China (0.17), and Australia (0.14). The United States ranked among the top in both output and centrality, indicating its pivotal role in knowledge dissemination and network connectivity. China ranked first in output but lower in centrality than in output ranking, suggesting that it primarily plays a “producer” rather than “hub” role. The 6th to 10th ranked countries were the United Kingdom (27, centrality 0.15), Germany (21, 0.34), France (16, 0.08), Turkey (12, 0.07), and Egypt (12, 0.01).

**Figure 4:**
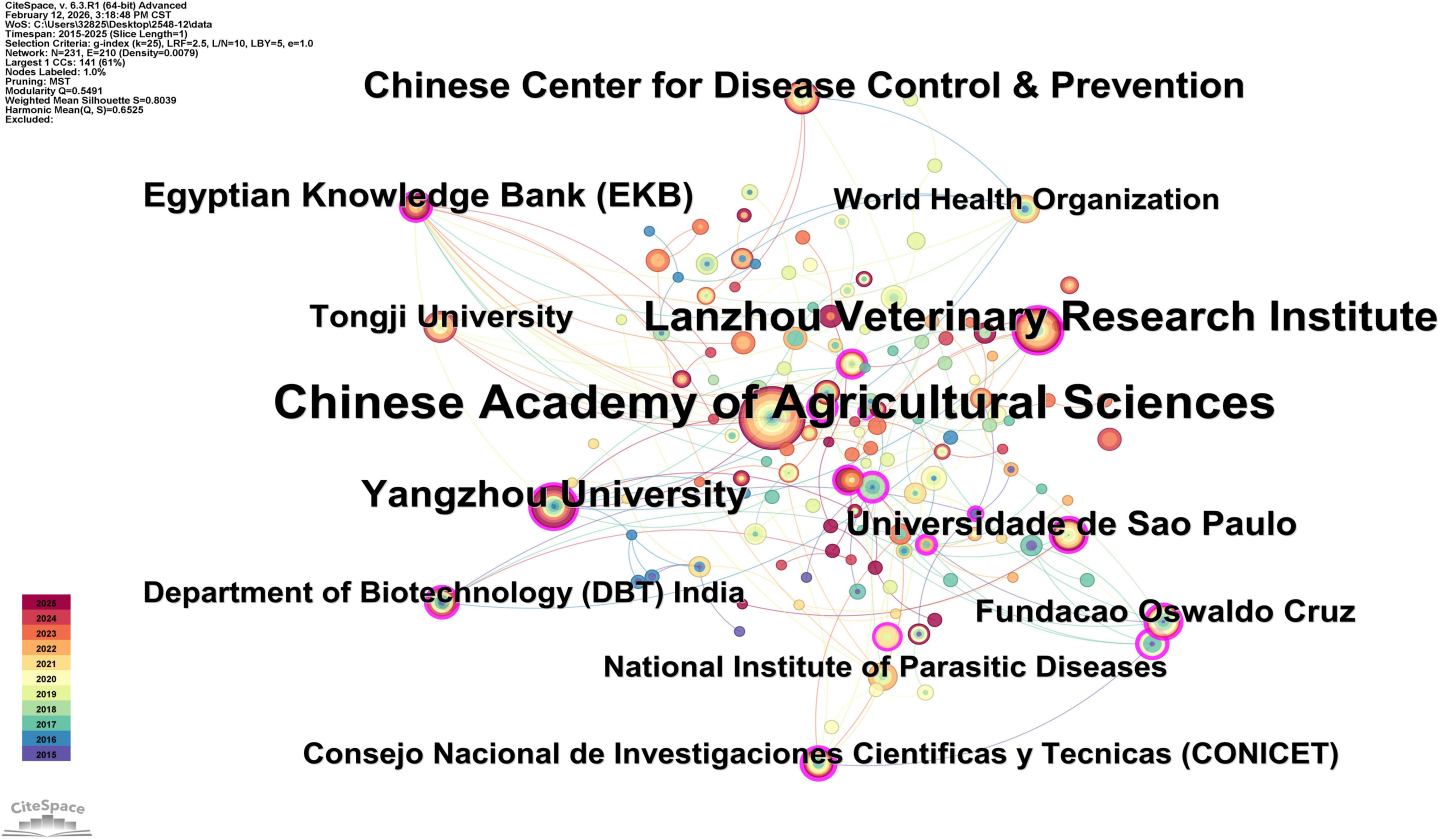
Institutional collaboration network. Nodes represent research institutions; node size reflects publication volume; link thickness represents collaboration strength.

In the institutional network (**Table 2**; **Figure 4**), the top five institutions by output were the Chinese Academy of Agricultural Sciences (36), Lanzhou Veterinary Research Institute (24), Yangzhou University (16), Chinese Center for Disease Control and Prevention (15), and Universidade de São Paulo (12). The top five by centrality were the European Molecular Biology Laboratory, Inserm, CONICET, Wellcome Trust Sanger Institute, and Lanzhou Veterinary Research Institute. The 6th to 10th ranked institutions were the Egyptian Knowledge Bank (12, centrality 0.15), Tongji University (9, 0.06), Fundação Oswaldo Cruz (9, 0.12), World Health Organization (8, 0.09), and National Institute of Parasitic Diseases (8, 0.03). The Lanzhou Veterinary Research Institute ranked highly in both output and centrality, indicating its dual role as a major research center and an important international collaboration hub.

**Table 2:** Top 10 institutions by publication volume and centrality.

Rank	Publication volume	Centrality	Publication year	Institution
1	36	0.06	2015	Chinese Academy of Agricultural Sciences
2	24	0.19	2016	Lanzhou Veterinary Research Institute
3	16	0.14	2016	Yangzhou University
4	15	0.01	2016	Chinese Center for Disease Control & Prevention
5	12	0.12	2017	Universidade de Sao Paulo
6	12	0.15	2015	Egyptian Knowledge Bank (EKB)
7	9	0.06	2019	Tongji University
8	9	0.12	2015	Fundacao Oswaldo Cruz
9	8	0.09	2016	World Health Organization
10	8	0.03	2018	National Institute of Parasitic Diseases

**Figure 5:**
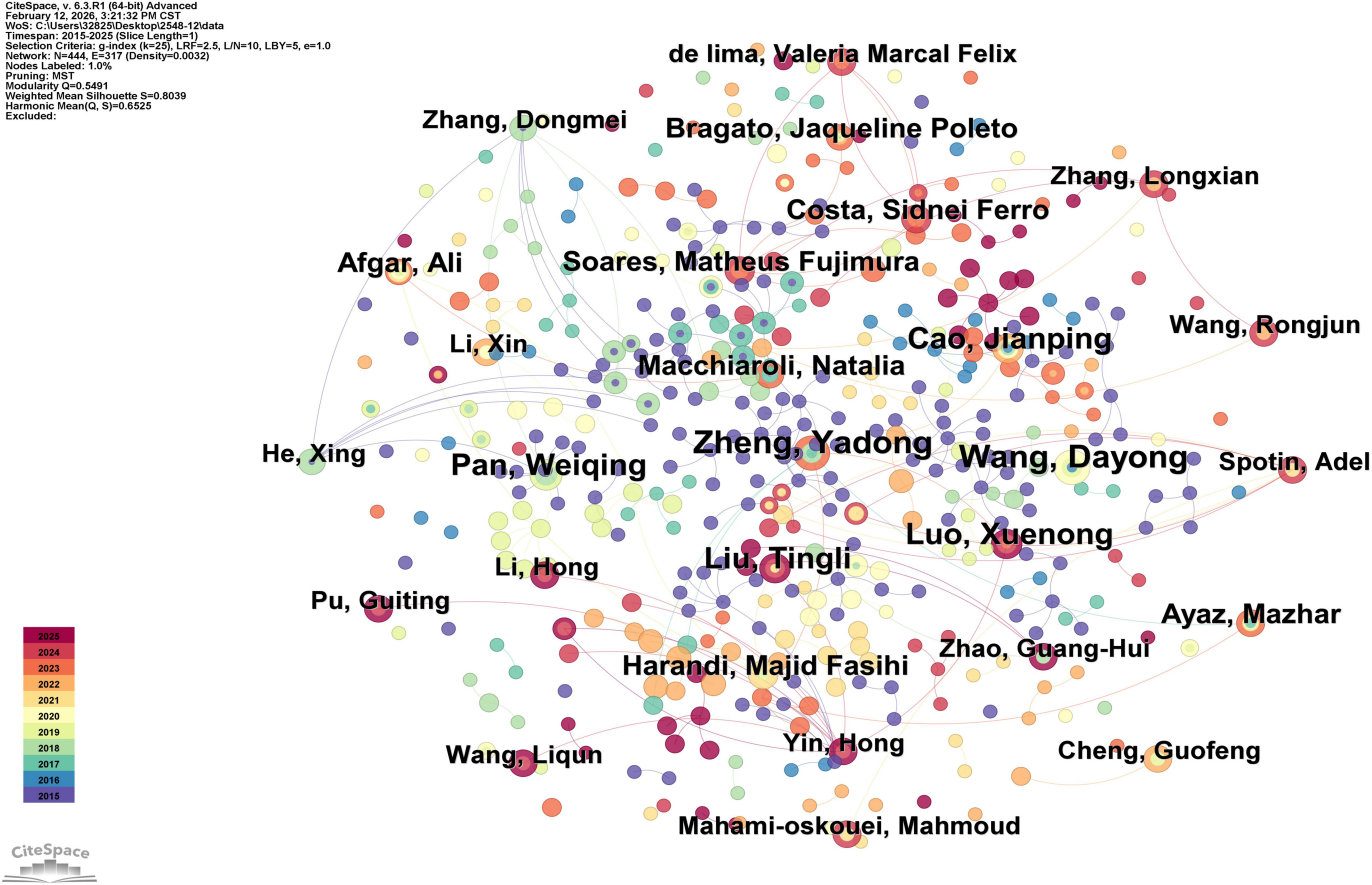
Author co−authorship network (N = 444, E = 317, density=0.0032). Nodes represent individual authors; node size reflects publication volume; links represent co−authorship relationships.

The author network consists of 444 nodes and 317 links, with a density of 0.0032 (**Table 3**; **Figure 5**). The most productive authors were Zheng, Yadong and Wang, Dayong (seven each), followed by Pan, Weiqing; Luo, Xuenong; Liu, Tingli; and Cao, Jianping (six each), and Soares, Bragato, Afgar, Costa, Harandi, and Macchiaroli (five each). The extremely low network density indicates that collaboration at the author level is relatively fragmented, and cross-team integration needs to be strengthened.

**Table 3:** Top 12 authors by publication volume.

Author	Publication volume	Country	Institution
Zheng, Yadong	7	China	Chinese Academy of Agricultural Sciences (CAAS)
Wang, Dayong	7	China	Southeast University
Pan, Weiqing	6	China	Naval Medical University
Luo, Xuenong	6	China	Chinese Academy of Agricultural Sciences (CAAS)
Liu, Tingli	6	China	Chinese Academy of Agricultural Sciences (CAAS)
Cao, Jianping	6	China	National Institute of Parasitic Diseases
Soares, Matheus Fujimura	5	Brazil	São Paulo State University (UNESP)
Bragato, Jaqueline Poleto	5	Brazil	São Paulo State University (UNESP)
Afgar, Ali	5	Iran	Kerman University of Medical Sciences
Costa, Sidnei Ferro	5	Brazil	São Paulo State University (UNESP)
Harandi, Majid Fasihi	5	Iran	Kerman University of Medical Sciences
Macchiaroli, Natalia	5	Argentina	University of Buenos Aires

### 3.3 Keyword clustering and evolutionary trends

**Figure 6:**
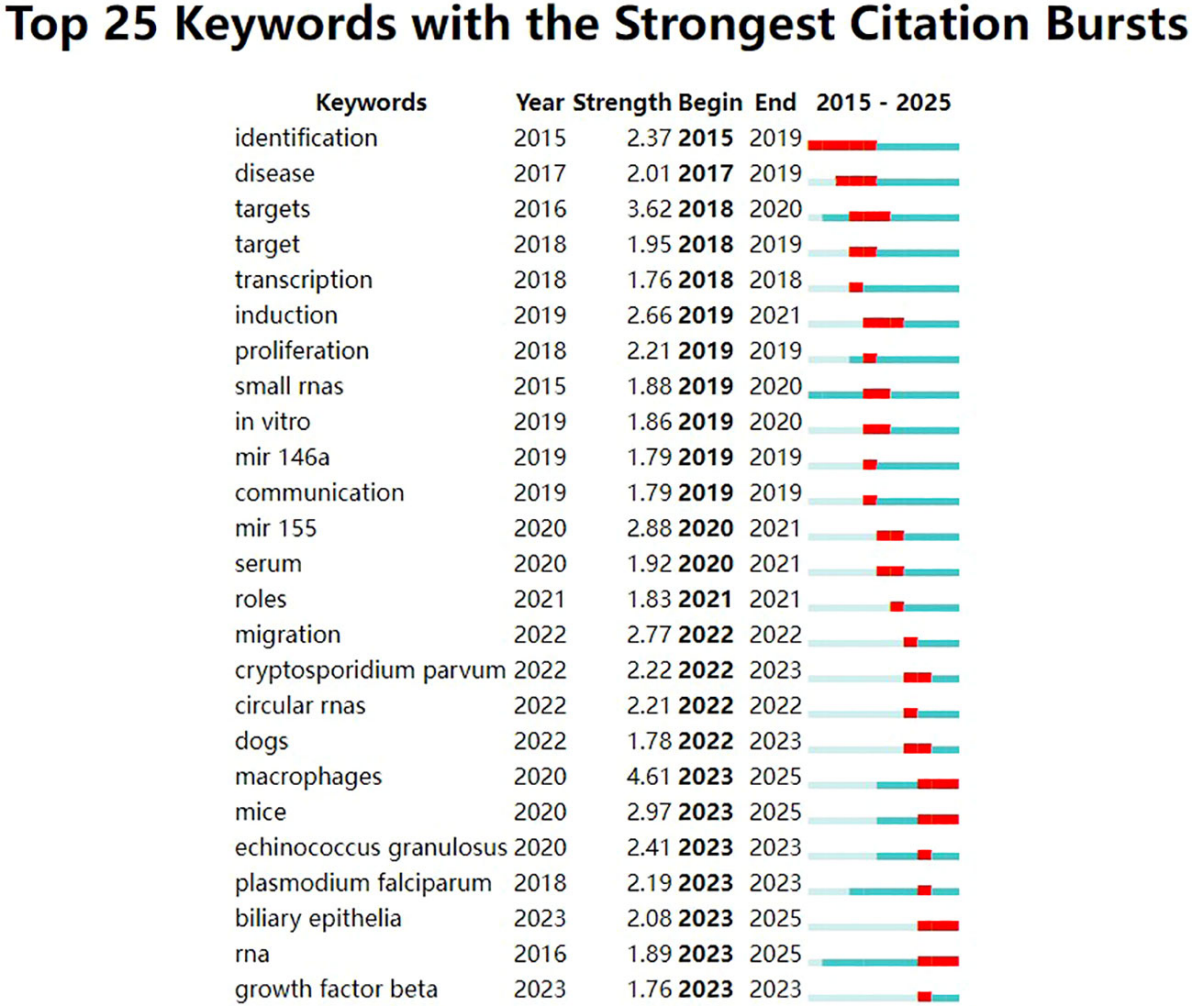
Keyword clustering map (Q = 0.5491, S = 0.8039). Ten major clusters (#0–#9) were identified using the LLR algorithm.

The keyword co-occurrence network consists of 325 nodes and 607 links, with a density of 0.0115. Cluster analysis identified 10 clusters (#0 to #9), with a modularity *Q* of 0.5491 and a weighted mean silhouette *S* of 0.8039 (**Table 4**; **Figure 6**). Major clusters included #0 liver (38 keywords, mean year 2017), #1 immune responses (36, 2020), #2 cystic echinococcosis (*Echinococcus*) (35, 2020), #3 extracellular vesicles (31, 2019), #4 hepatic fibrosis (30, 2019), #5 *Toxoplasma gondii* (29, 2020), #6 *Anopheles gambiae* (28, 2018), #7 disease (25, 2019), #8 gene expression (24, 2021), and #9 genome (23, 2017).

**Table 4:** Summary of keyword clusters.

Cluster ID	Size	Silhouette	Mean (year)	Label (LLR)
0	38	0.793	2017	liver; immune response; science; immunosuppression; cardiomyopathy
1	36	0.745	2020	immune responses; immune response; visceral leishmaniasis; mir-155; leishmania major
2	35	0.787	2020	cystic echinococcosis; liver fibrosis; alveolar echinococcosis; nox2; e. multilocularis
3	31	0.825	2019	extracellular vesicles; nosema ceranae; apis cerana cerana; resistance; macrophage
4	30	0.797	2019	hepatic fibrosis; schistosoma japonicum; mansoni; vector; reveals
5	29	0.76	2020	toxoplasma gondii; forkhead box p3; bgtep1; avirulent and virulent strains; biomphalaria glabrata
6	28	0.819	2018	anopheles gambiae; rna; mosquito reproduction; microrna expression; development
7	25	0.855	2019	disease; cryptosporidium parvum; hct-8 cell; parasite burden; long non-coding rna
8	24	0.793	2021	gene expression; macrophages; donovani; dna methylation; post-kala-azar dermal leishmaniasis
9	23	0.832	2017	genome; d glucosyl hydroxymethyluracil; antigenic variation; surface glycoprotein genes; cardiac cellular model

**Figure 7:**
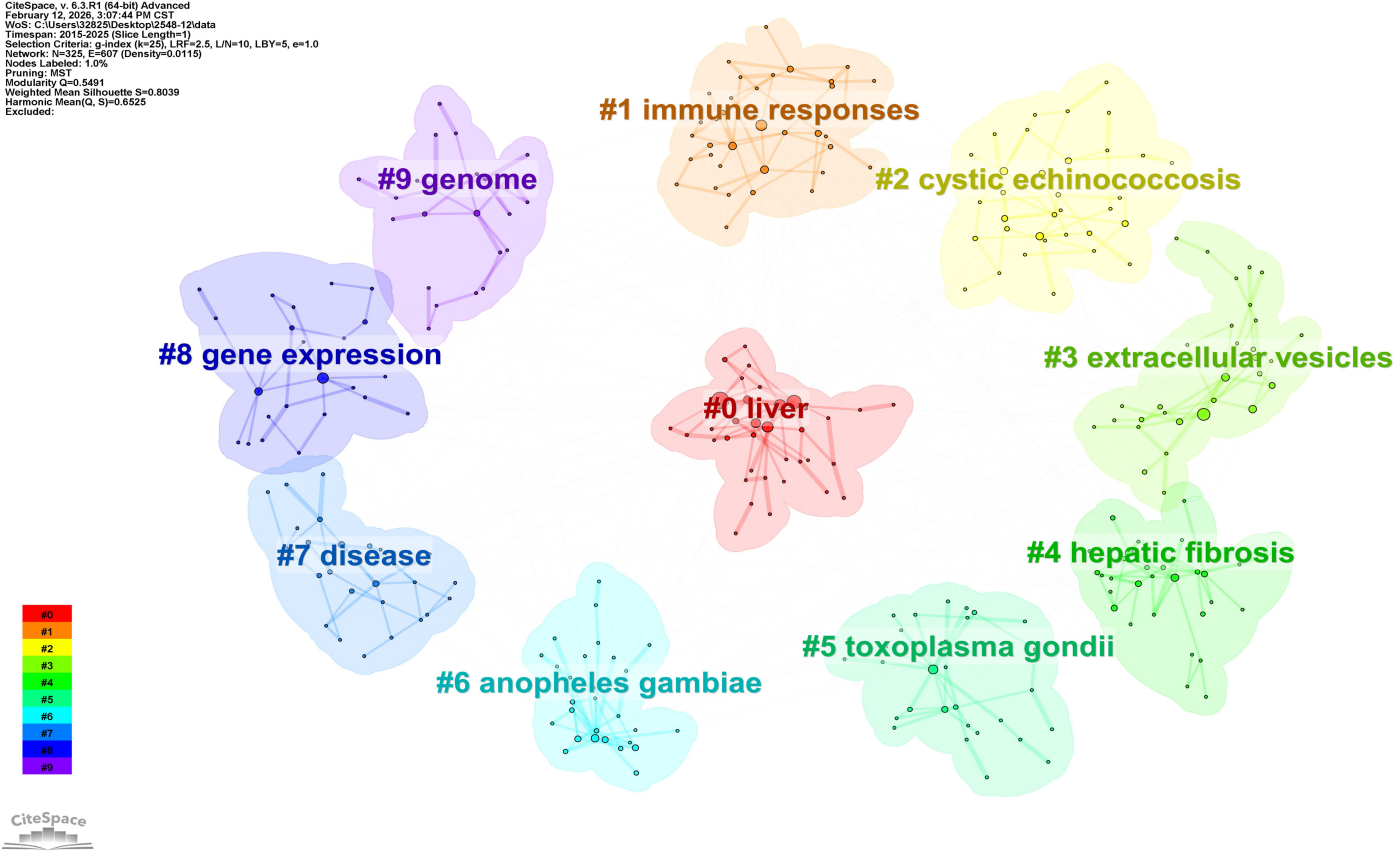
Timeline view of keyword clusters showing the temporal evolution of research hotspots (2015–2025). Each horizontal line represents a cluster; node positions indicate the year of keyword appearance; node size reflects frequency.

The timeline evolution map (**Figure 7**) further revealed the temporal dynamics of each cluster. #0 liver and #1 immune responses exhibited node distribution across every year from 2015 to 2025, persisting throughout the entire study period and indicating that these two themes remained consistent focal points of the field. #3 extracellular vesicles showed sparse node distribution prior to 2018 but a sharp increase in node density thereafter, with the cluster rapidly expanding to 31 keywords and establishing connections with #0, #1, #4, and #8, indicating that this theme emerged as a rising hotspot after 2018 and became a key node linking immune regulation, hepatic fibrosis, and gene expression. #8 gene expression had the latest mean year (2021), suggesting sustained activity in this research theme in recent years. *NF-κB* appeared across multiple clusters, suggesting that this classical inflammatory pathway occupies a central hub position in the ncRNA regulatory network.

**Figure 8:**
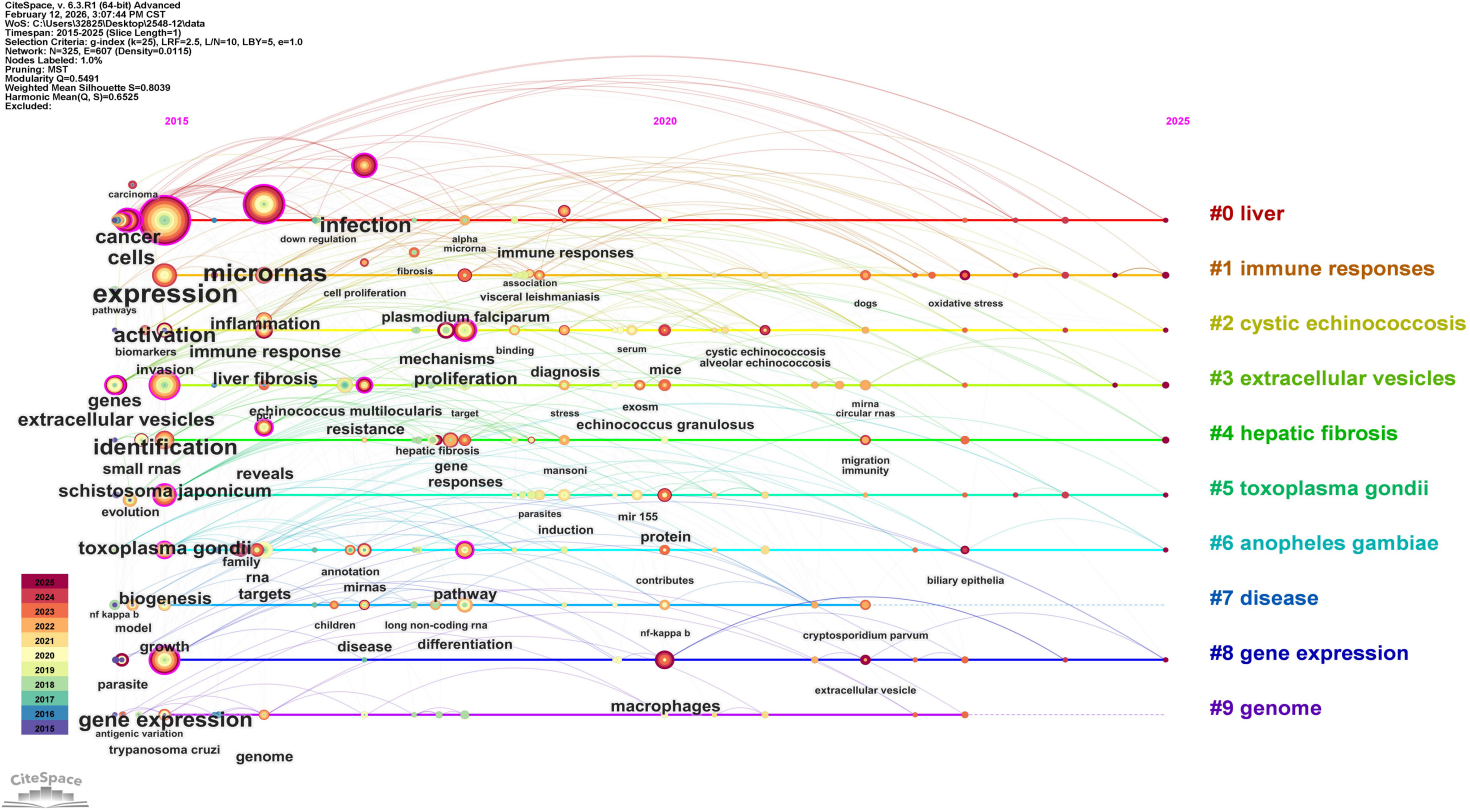
Top 25 keywords with the strongest citation bursts (2015–2025). The blue line represents the overall time span; the red bar indicates the burst duration of each keyword.

Keyword burst detection identified the top 25 keywords by burst intensity (**Figure 8**). The top three were “macrophages” (4.61, 2023–2025), “targets” (3.62, 2018–2020), and “mice” (2.97, 2023–2025). Temporally, keywords that burst from 2015–2019 included “identification,” “disease,” and “targets,” primarily focusing on pathogen identification and disease target exploration; from 2019–2021 included “induction,” “miR-146a,” “miR-155,” and “communication,” reflecting a shift toward ncRNA regulatory and cellular communication mechanisms; from 2022–2025 included “*Cryptosporidium parvum*,” “circular RNAs,” “macrophages,” “mice,” and “biliary epithelia,” centering on specific pathogens, circular RNAs, and host immune interactions. This three-phase evolution, together with the rapid rise of cluster #3 observed in the timeline map, collectively delineates a clear trajectory from molecular identification to functional regulation and further to host–pathogen interactions.

### 3.4 Reference co-citation analysis

**Figure 9:**
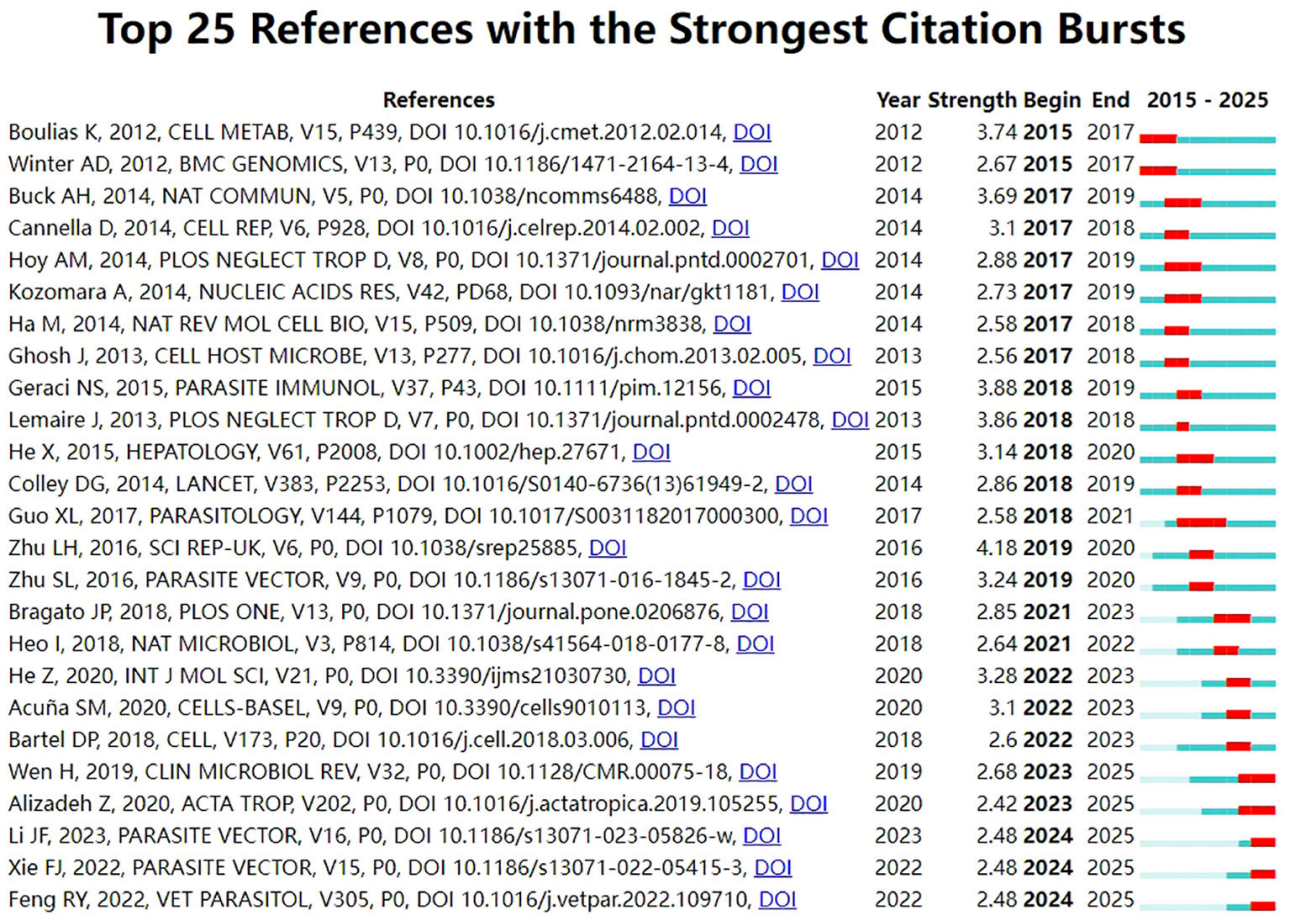
Top 25 references with the strongest citation bursts (2015–2025). The blue line represents the overall time span; the red bar indicates the burst duration of each reference.

Reference co-citation analysis identified the most frequently cited references in the field (**Table 5**; **Figure 9**). The most cited work was by Mantel et al. (2016, 186 citations) on *Plasmodium* extracellular vesicles delivering miRNAs to host endothelial cells, followed by Broadbent et al. (2015, 141) on systematic annotation of lncRNAs and circRNAs in *Plasmodium*, Lambertz et al. (2015, 125) on functional miRNA activity in *Leishmania* extracellular vesicles, Liu et al. (2019, 116) on *Schistosoma japonicum* extracellular vesicle miRNA cargo regulating host macrophage functions, and Samoil et al. (2018, 113) on protein and miRNA content of *Schistosoma mansoni* exosome-like vesicles. The 6th to 10th most cited references were Atayde et al. (2019, 93), Muxel et al. (2017, 78), Geraci et al. (2015, 77), Frank et al. (2015, 68), and He et al. (2015, 67). These highly cited references collectively established the research paradigm of “extracellular vesicles–non-coding RNAs as core messengers in host–parasite interactions”.

**Table 5:** Top 10 most cited references.

Ranking	Title	Author	Year	Count	Journal
1	Infected erythrocyte-derived extracellular vesicles alter vascular function via regulatory Ago2-miRNA complexes in malaria	Mantel, PY	2016	186	NATURE COMMUNICATIONS
2	Strand-specific RNA sequencing in Plasmodium falciparum malaria identifies developmentally regulated long non-coding RNA and circular RNA	Broadbent, KM	2015	141	BMC GENOMICS
3	Small RNAs derived from tRNAs and rRNAs are highly enriched in exosomes from both old and new world Leishmania providing evidence for conserved exosomal RNA Packaging	Lambertz, U	2015	125	BMC GENOMICS
4	Schistosoma japonicum extracellular vesicle miRNA cargo regulates host macrophage functions facilitating parasitism	Liu, JT	2019	116	PLOS PATHOGENS
5	Vesicle-based secretion in schistosomes: Analysis of protein and microRNA (miRNA) content of exosome-like vesicles derived from Schistosoma mansoni	Samoil, V	2018	113	SCIENTIFIC REPORTS
6	Exploitation of the Leishmania exosomal pathway by Leishmania RNA virus 1	Atayde, VD	2019	93	NATURE MICROBIOLOGY
7	Leishmania (Leishmania) amazonensis induces macrophage miR-294 and miR-721 expression and modulates infection by targeting NOS2 and L-arginine metabolism	Muxel, SM	2017	78	SCIENTIFIC REPORTS
8	Characterization of microRNA expression profiles in Leishmania-infected human phagocytes	Geraci, NS	2015	77	PARASITE IMMUNOLOGY
9	Autophagic digestion of Leishmania major by host macrophages is associated with differential expression of BNIP3, CTSE, and the miRNAs miR-101c, miR-129, and miR-210	Frank, B	2015	68	PARASITES & VECTORS
10	Recombinant adeno-associated virus-mediated inhibition of microRNA-21 protects mice against the lethal schistosome infection by repressing both IL-13 and transforming growth factor beta 1 pathways	He, X	2015	67	HEPATOLOGY

Five references exhibited citation bursts continuing to 2025: Wen et al. (2019); Alizadeh et al. (2020); Li et al. (2023); Xie et al. (2022), and Feng et al. (2022), primarily focusing on clinical management of echinococcosis, diagnostic biomarkers, and ncRNA research in veterinary parasitology, indicating that the field is accelerating its transition from fundamental discoveries toward clinical applications.

### 3.5 Journal dual-map overlay

**Figure 10:**
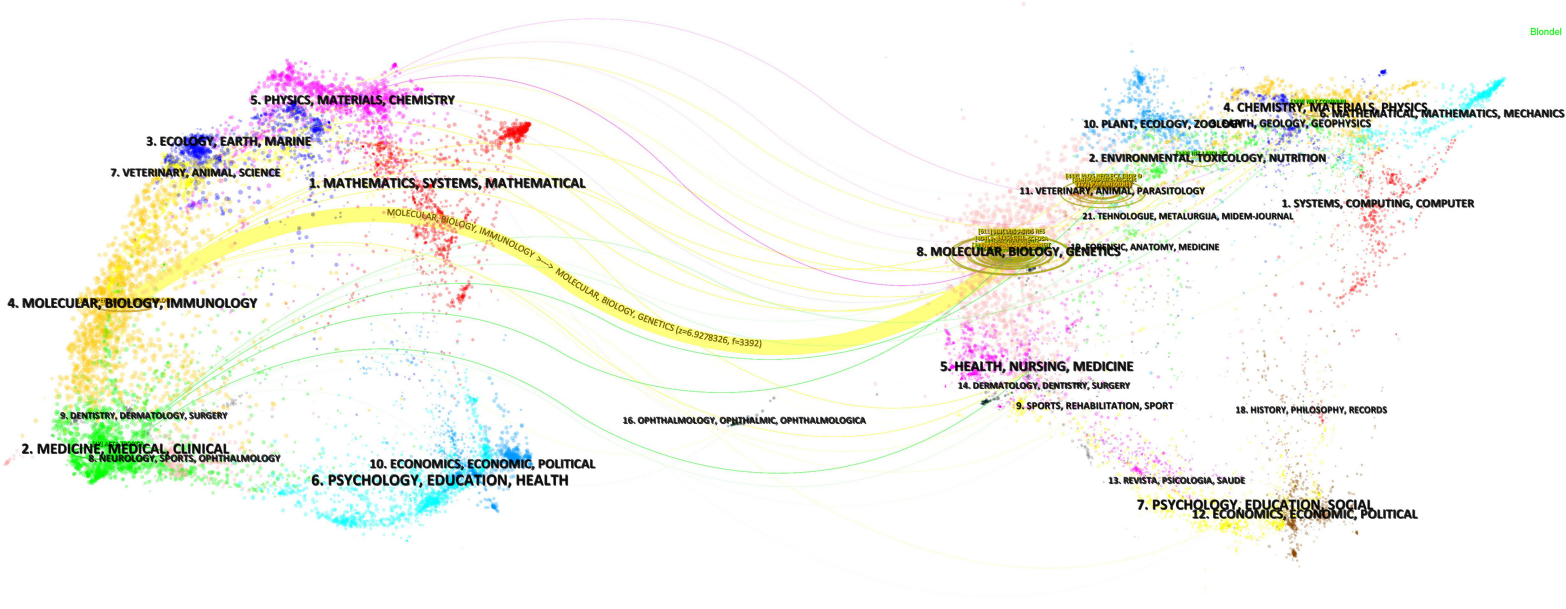
Dual−map overlay of citing and cited journals. The left side represents citing journals; the right side represents cited journals. Colored paths indicate citation flows between disciplines.

The journal dual-map overlay in **Figure 10** reveals the distinct interdisciplinary characteristics of knowledge flow within this research field. The citing journals on the left are primarily centered in Cluster 4 (Molecular Biology and Immunology), with Cluster 3 (Economy, Earth, and Marine) and Cluster 5 (Physics, Materials, and Chemistry) also serving as significant citing sources. The yellow and green core citation trajectories, originating from left Cluster 4, point respectively to right Cluster 8 (Molecular Biology and Genetics) and Cluster 11 (Veterinary Medicine, Animal Science, and Parasitology). This not only underscores the field’s theoretical foundation in molecular mechanisms but also demonstrates its active translational expansion toward applied veterinary sciences. Furthermore, connections from left Cluster 3 extend to right Cluster 10 (Plant, Ecology, Zoology, Geology, and Geophysics), while left Cluster 5 connects to right Cluster 4 (Chemistry, Materials, and Physics) and Cluster 1 (Systems, Computing, and Computer). These multidimensional links provide robust evidence that theories and methodologies from diverse disciplines, including environmental ecology and mathematical computing, are increasingly integrated into this research framework.

## 4 Discussion

### 4.1 Research landscape and intellectual base

From 2015 to 2025, publications in the field of non-coding RNAs in parasitic diseases increased from 21 to 409, with a net growth of 388 articles over the decade—a more than 19−fold increase. This sustained expansion is closely linked to the widespread adoption of high-throughput sequencing technologies—from early miRNA microarrays to whole-transcriptome sequencing, technological advances have enabled systematic identification of ncRNA molecules across an increasing number of parasite species (Broadbent et al., 2015), and have shifted research perspectives from single-molecule discovery toward regulatory network analysis. However, the continued growth also suggests that the field has not yet reached saturation, with core scientific questions—such as how ncRNAs dynamically regulate gene expression across different parasite life stages and the mechanisms governing selective packaging of RNA cargo in cross-kingdom communication—remain unanswered (Guegan et al., 2022; Pu et al., 2025).

Regarding international collaboration patterns, China ranks first in publication output (175 articles) but ranks lower in centrality than in output ranking. The United States, with approximately one-third of China’s output (64 articles), ranks first in centrality (0.37), while Germany (0.34) and Spain (0.29) also exhibit centrality exceeding their output rankings. This pattern suggests that high-output countries are not necessarily high-influence hubs. China’s research in this field is characterized by large-scale output but relatively limited capacity for knowledge dissemination and coordination within the international collaboration network. At the author level, the collaboration network is extremely sparse (0.0032), reflecting the fragmented nature of research forces in this field, which may be associated with the geographical distribution of parasitic diseases and disparities in research resources.

Highly cited reference analysis reveals that the most frequently cited studies all revolve around the core paradigm of “extracellular vesicles–non-coding RNAs,” collectively establishing the intellectual foundation of this field. Mantel et al. (2016, 186 citations) first demonstrated that Plasmodium-infected erythrocyte-derived EVs deliver Ago2-miRNA complexes to host endothelial cells, modulating vascular barrier function (Mantel et al., 2016). Broadbent et al. (2015, 141 citations) established the first transcriptome-wide ncRNA atlas in Plasmodium falciparum by identifying over one thousand lncRNAs and hundreds of circRNAs (Broadbent et al., 2015). Lambertz et al. (2015, 125 citations) extended this paradigm by showing that Leishmania EVs are enriched with tRNA- and rRNA-derived small RNAs that can be delivered to host cells (Lambertz et al., 2015). Subsequent studies applied this framework to other parasites: Liu et al. (2019, 116 citations) demonstrated that S. japonicum EV miRNAs (miR-125b and bantam) enter host macrophages and modulate proliferation and TNF-α production via Pros1, Fam212b, and Clmp to facilitate parasite survival (Liu et al., 2019); Samoil et al. (2018, 113 citations) reported that Schistosoma EVs contain over 140 miRNAs, 25 of which are detectable in infected host serum (Samoil et al., 2018), supporting their diagnostic potential. Notably, He et al. (2015, 67 citations) showed that miR-21 is upregulated in hepatic stellate cells via IL-13/TGF-β1–SMAD signaling during schistosomiasis, and AAV8-mediated miR-21 inhibition attenuated hepatic fibrosis by enhancing SMAD7 expression (He et al., 2015). Collectively, these studies confirm that parasite-derived ncRNAs function not merely as communication signals but as effector molecules with defined pathological roles.

References with citation bursts continuing to 2025 further indicate the direction of translational applications. Wen et al. (2019) published a comprehensive review on echinococcosis in *Clinical Microbiology Reviews*, systematically summarizing the epidemiology, diagnosis, and treatment advances of the disease (Wen et al., 2019). Alizadeh et al. (2020) systematically evaluated the potential of *Echinococcus*-specific miRNAs in serum as diagnostic biomarkers (Alizadeh et al., 2020). Li et al. (2023); Xie et al. (2022), and Feng et al. (2022) explored the application of parasite-derived miRNAs in early diagnosis of cysticercosis and echinococcosis from different perspectives (Feng et al., 2022; Li et al., 2023; Xie et al., 2022). These persistently active references collectively indicate that the field is accelerating its transition from fundamental discoveries toward clinical applications.

### 4.2 Stage-specific evolution of frontier themes

Keyword clustering analysis identified 10 clusters, among which "liver" and "immune responses" persisted throughout the entire study period—a pattern highly consistent with the pathological features of parasitic diseases such as echinococcosis and schistosomiasis, which both affect the liver as the primary organ involved and depend on immune responses as the core determinant of infection outcomes. "Hepatic fibrosis" emerged as an independent cluster, reflecting systematic attention to the regulatory roles of ncRNAs in parasite-induced hepatic fibrosis. Wang et al. (2017) confirmed that miR-21 promotes hepatic stellate cell activation by targeting Smad7 in schistosomiasis-associated hepatic fibrosis (Wang et al., 2017), while He et al. (2020) revealed the mechanism by which *Schistosoma*-derived miRNAs cross-kingdom promote hepatic fibrosis (He et al., 2020). These two lines of research represent, respectively, host endogenous miRNAs and parasite-derived miRNAs in the regulation of hepatic fibrosis, suggesting that host- and parasite-derived ncRNAs may synergistically drive pathological progression. This "collusion from both sides" regulatory mode may be a key feature distinguishing parasite-induced hepatic fibrosis from other types of liver fibrosis. "Extracellular vesicles" rose rapidly after 2018 as a key node connecting various clusters, corroborating the broad influence of the EVs-ncRNA research paradigm.

Keyword burst analysis revealed a three-phase evolutionary trajectory. The first phase (2015–2019), characterized by "identification" and "targets," corresponds to the early molecular identification work. Researchers during this phase primarily focused on discovering ncRNA molecules present in different parasite species and their predicted target genes (Broadbent et al., 2015), representing a "resource accumulation period" for the field. The second phase (2019–2021), marked by "miR-146a," "miR-155," and "communication," signified a shift from molecular discovery toward functional validation and communication mechanisms. Multiple immune-regulatory miRNAs emerged intensively during this period, and the simultaneous burst of “communication” suggests that researchers’ understanding of the role of ncRNAs in intercellular communication began to take shape. In the context of parasitic infections, the immune functions of miR-146a and miR-155 became focal points—Rego et al. (2023) found that miR-146a showed consistent responsiveness across epithelial cells, cardiomyocytes, and macrophages infected with Trypanosoma cruzi, suggesting its broad involvement in host defense (Rego et al., 2023).

The third phase (2022 -2025) is distinctly marked by a shift toward pathogen-specific immune microenvironments and mechanistic hubs, as reflected in the sustained bursts of "macrophages", "circular RNAs", "biliary epithelia", and "NF-κB" --indicating that the field has moved from generic functional studies into disease-contextualized networks and translational exploration. Consistent with this phase, the high burst intensity of "macrophages" (4.61) reflects the central role of macrophages as the primary host cells for multiple parasites (e.g., *Leishmania*, *Toxoplasma*, *Schistosoma*). Macrophage polarization status determines infection outcomes—M1 macrophages promote parasite clearance, while M2 macrophages facilitate parasite survival and tissue repair. Sais et al. demonstrated that both host and parasite non-coding RNAs coordinately regulate macrophage gene expression during *Fasciola hepatica* infection, reducing pro-inflammatory responses and promoting tissue repair pathways (Sais et al., 2024); Liu et al. revealed that *Toxoplasma* negatively regulates host immune signaling by inducing differential expression of host lncRNAs (Liu et al., 2018). These findings collectively suggest that the ncRNA–macrophage axis may be one of the core pathways through which parasites modulate the host immune microenvironment.

Also falling within this third phase, the emergence of "biliary epithelia" as a new burst keyword during 2023 -2025 carries significant biological implications, as biliary epithelial cells (cholangiocytes) are the primary target cells for liver fluke infections (*Clonorchis sinensis*, *Opisthorchis viverrini*) and *Cryptosporidium* biliary infections. Chen et al. demonstrated that Plasmodium can modulate host long non-coding RNA expression during erythrocytic infection (Chen et al., 2022), suggesting that ncRNAs play a general role in parasite–epithelial cell interactions. The ncRNA regulatory network in biliary epithelial cells may be closely associated with bile duct fibrosis and cholangiocarcinogenesis induced by liver fluke infection, making this a direction worthy of in-depth investigation. In the context of biliary epithelia-related parasitic diseases, the granulomatous response and hepatic fibrosis induced by schistosome egg deposition in the liver represent the most significant pathological changes. Zhao et al. (2019) demonstrated that mmu-miR-92a-2-5p alleviates schistosomiasis-induced hepatic fibrosis by targeting TLR2, suggesting an important role of ncRNAs in regulating hepatic granulomatous responses (Zhao et al., 2019).

circRNAs represent another important frontier. The earliest evidence for circRNAs in parasites came from Broadbent et al., who not only identified a large number of lncRNAs but also predicted hundreds of circRNAs in *Plasmodium falciparum* through strand-specific RNA sequencing (Broadbent et al., 2015). In recent years, research on circRNAs in parasitic diseases has made significant progress. Yin et al. revealed that host circRNA ciRS-7 promotes *Cryptosporidium parvum* proliferation by sponging miR-1270 to activate the NF-κB signaling pathway (Yin et al., 2021). Liu et al. constructed circRNA-miRNA-mRNA interaction networks in hepatocytes following *Echinococcus multilocularis* infection (Liu et al., 2022). Dai et al. further demonstrated that circGsr-0002 promotes schistosomiasis-induced hepatic fibrosis by regulating the DNMT3A/PTEN pathway (Dai et al., 2025). Collectively, these studies indicate that circRNAs, with their nuclease resistance conferred by circular structure and their potential function as miRNA sponges, have demonstrated significant value in biomarker discovery and regulatory network studies in parasitic diseases.

NF-κB appears across multiple clusters, suggesting that this classical inflammatory pathway occupies a central hub position in the ncRNA regulatory network. Yin et al. demonstrated that ciRS-7 activates the NF-κB pathway by sponging miR-1270 (Yin et al., 2021). Zheng (2018) found that *Echinococcus* infection downregulates host miR-222-3p to regulate NF-κB-related genes (CD14, TLR4, TICAM2, AP-1) in the LPS/TLR4 pathway (Zheng, 2018). Zhang et al. (2020) confirmed that *Cryptosporidium parvum* upregulates host miR-942-5p via TLR2/TLR4-NF-κB signaling. These findings suggest that future research may further reveal how ncRNAs fine-tune inflammatory responses by targeting different components of the NF-κB pathway (Zhang et al., 2020).

### 4.3 Technology-driven translational trends

Technological iteration is the primary driver of progress in this field. From bulk RNA-seq to single-cell sequencing and spatial transcriptomics, increasing technical resolution enables researchers to dissect the heterogeneity of ncRNA expression in parasites across different host tissue microenvironments. Broadbent et al. achieved whole-transcriptome identification of ncRNAs in *Plasmodium* through strand-specific RNA sequencing (Broadbent et al., 2015), representing the first systematic ncRNA atlas construction in this field. Gillan et al. combined transcriptomic and miRNA expression profiling to reveal extracellular vesicle miRNA and protein cargo characteristics in *Theileria annulata*-infected leukocytes (Gillan et al., 2019). Ikoma et al., in the complex context of malaria co-infected with KSHV, revealed significant changes in exosomal miRNA expression profiles through small RNA sequencing (Ikoma et al., 2018). However, the high proportion of repetitive sequences and incomplete annotations in parasite genomes still pose challenges for accurate ncRNA identification. Yuan et al. achieved *in vivo* blockade of host miR-101b-3p using AAV-delivered TuD inhibitors (Yuan et al., 2019), providing a technical example for functional studies of ncRNAs in parasitic diseases. The journal dual-map overlay shows knowledge flow extending from basic molecular biology toward veterinary medicine and parasitology, with ecology, physics, and mathematics clusters also appearing among cited journals, reflecting the infiltration of bioinformatics and computational biology methodologies.

In terms of translation, parasite-derived circulating miRNAs as non-invasive diagnostic markers represent the most promising near-term application direction (Li et al., 2025). For diseases such as echinococcosis that currently rely on imaging and invasive examinations, detection of parasite-specific miRNAs in serum or plasma could enable early diagnosis and treatment monitoring (Alizadeh et al., 2020; Wen et al., 2019). Furthermore, Wang et al. developed a rolling circle amplification-assisted CRISPR/Cas9-based detection method that achieves highly sensitive detection of let-7-5p from cysticercosis and echinococcosis (with a limit of detection of 10 aM and diagnostic sensitivity and specificity of 100% and 97.67%, respectively) (Wang et al., 2024), providing a technical example for translating ncRNA diagnostics from the laboratory to clinical practice. Extracellular vesicles as natural nanocarriers also show potential for anti-parasitic nucleic acid drug delivery—EVs can protect their RNA cargo from nuclease degradation and deliver functional miRNAs to target cells (Rego et al., 2023). Pourabbasi Ardekan et al. (2025) further explored the application of miR-155 chitosan polyplex as an immunoadjuvant in the treatment of leishmaniasis, offering new perspectives for ncRNA-based therapeutic strategies (Pourabbasi Ardekan et al., 2025). However, the journey from laboratory discovery to clinical application still requires overcoming obstacles including assay standardization, large-scale clinical cohort validation, and cost control.

This study has several limitations. Bibliometric analysis itself has citation bias—highly cited references do not necessarily equate to higher scientific quality. The selection of CiteSpace analysis parameters (such as the k value of g-index and MST pruning) may influence network structure, and different parameter settings may yield slightly different results. Additionally, this study only included original research articles, potentially omitting some important opinion-based content.

## 5 Conclusions

Through bibliometric analysis, this study systematically examined the research landscape and evolutionary trends of non-coding RNAs in parasitic diseases from 2015 to 2025. The field has demonstrated sustained growth over the past decade, with research priorities shifting from early molecular identification toward functional regulation and cross-kingdom communication mechanisms. China ranks first in global publication output in this field, while the United States exhibits the strongest centrality in the international collaboration network; the sparse author-level collaboration structure highlights an urgent need for enhanced cross-team integration. Keyword analysis identified hepatic pathology and immune regulation as persistent core themes, with extracellular vesicles emerging as a critical node connecting multiple research dimensions. Highly cited references established the paradigm of “EVs-ncRNA as core messengers in host–parasite interactions,” while persistently bursting references point toward clinical diagnostic and translational applications. Future research should focus on elucidating the molecular mechanisms of cross-kingdom communication, functional characterization of novel ncRNAs and their translational potential, and promote sustained advancement through enhanced international collaboration and interdisciplinary integration.

## Data Availability

The raw data supporting the conclusions of this article will be made available by the authors, without undue reservation.
